# The Role and Mechanism of Lysine Methyltransferase and Arginine Methyltransferase in Kidney Diseases

**DOI:** 10.3389/fphar.2022.885527

**Published:** 2022-04-26

**Authors:** Xun Zhou, Hui Chen, Jinqing Li, Yingfeng Shi, Shougang Zhuang, Na Liu

**Affiliations:** ^1^ Department of Nephrology, Shanghai East Hospital, Tongji University School of Medicine, Shanghai, China; ^2^ Department of Medicine, Rhode Island Hospital and Alpert Medical School, Brown University, Providence, RI, United States

**Keywords:** epigenetic modification, lysine methyltransferase, arginine methyltransferase, acute kidney injury, chronic kidney diseases, renal cell carcinoma

## Abstract

Methylation can occur in both histones and non-histones. Key lysine and arginine methyltransferases under investigation for renal disease treatment include enhancer of zeste homolog 2 (EZH2), G9a, disruptor of telomeric silencing 1-like protein (DOT1L), and protein arginine methyltransferases (PRMT) 1 and 5. Recent studies have shown that methyltransferases expression and activity are also increased in several animal models of kidney injury, such as acute kidney injury(AKI), obstructive nephropathy, diabetic nephropathy and lupus nephritis. The inhibition of most methyltransferases can attenuate kidney injury, while the role of methyltransferase in different animal models remains controversial. In this article, we summarize the role and mechanism of lysine methyltransferase and arginine methyltransferase in various kidney diseases and highlight methyltransferase as a potential therapeutic target for kidney diseases.

## Introduction

Epigenetics refers to heritable changes in gene function and phenotype without altering the gene’s DNA sequence (Berger et al., 2009). There is variability in epigenetics with the onset and progression of diseases compared with inherited genetic variation, which provides an opportunity to use epigenetic changes as biomarkers for diseases (Rozek et al., 2014). Therefore, targeting associated epigenetic factors with small molecules may provide an effective way to “repair” disordered gene/chromosomal regulatory systems caused by abnormal epigenetic profiles. DNA methylation, histone modification, chromatin recombination and non-coding RNA are the main ways of Epigenetic regulation (Rosner and Hengstschläger, 2012; Roy and Majumdar, 2012). These modifications are closely related to the normal growth and development of individuals, and the occurrence and development of cancer, diabetes, heart disease, kidney disease and other diseases. The importance and diversity of histone post-translational modifications in epigenetic regulation have been widely studied. Methylation is one of the ways in which histones are modified, and can occur in both histones and non-histones, including transcription factors and chromatin modifiers (Worden et al., 2019). Unlike histone acetylation, histone methylation does not change the charge of lysine, but alters gene transcription by providing binding sites for chromosomal modifications (Thakore et al., 2016). The regulation effect of histone methylation and demethylation on gene transcription is mainly catalyzed by histone methyltransferase and demethylase (Thakore et al., 2016). Histones are proteins that are abundant in lysine and arginine, and their methylations occurring mainly at arginine (R) and lysine (K) residues in their N-terminal tails (Wei et al., 2009). Lysine methylation includes mono-methylation, di-methylation and tri-methylation. Arginine methylation includes mono-methylation and di-methylation (Di Lorenzo and Bedford, 2011). Key lysine and arginine methyltransferases under investigation for renal disease treatment include EZH2, G9a, DOT1L, PRMT1 and PRMT5 (Rugo et al., 2020). In recent years, histone methyltransferases have become the target of a series of small molecule inhibitors in the aera of kidney diseases. The development of the histone methyltransferases is discussed in the following paragraphs.

## Lysine Methyltransferase

Lysine methylation imparts active or repressive transcription depending on its location and methylation state (Black et al., 2012). In general, histone H3 at lysine 4 (H3K4), lysine 36 (H3K36), and lysine 79 (H3K79) methylation are considered markers of active transcription, while histone H3 at lysine 9 (H3K9), lysine 27 (H3K27), and histone H4 at lysine 20 (H4K20) methylation are thought to be associated with silenced chromatin status (Black et al., 2012). The large number of methylation sites and differential methylation states in histones suggest the complexity of the regulation of this signaling system and its dysregulation might play an important role in the occurrence and development of diseases. The role of three histone methyltransferases with H3K27, H3K79 and H3K9 as substrates in renal diseases will be discussed below.

### Enhancer of Zeste Homolog 2

EZH2 is a histone lysine methyltransferase that forms a catalytic subunit of PRC2 complex. It primarily catalyzes H3K27 methylation, which leads to gene silencing (Fioravanti et al., 2018). In recent years, its role in AKI and chronic kidney disease (CKD) has attracted more and more attention. AKI is a common disease which can be caused by drugs, intoxication, infection, ischemia reperfusion (IR), rhabdomyolysis and other causes (Bouchard et al., 2015). The incidence of AKI is 5.0–7.5% in general hospitalized patients and up to 50–60% in severe patients (Yoon et al., 2022). The current diagnostic criteria for AKI is a sharp decrease in glomerular filtration rate (GFR), as shown by a sharp increase in serum creatinine levels or a decrease in urine volume during the fixed period (Yoon et al., 2022). Due to increasing attention and continuous improvement of clinical diagnosis and treatment technology, the risk of death is decreasing, but long-term follow-up data shows that about 1/4 of AKI patients will evolve into CKD (Noble et al., 2020). CKD is characterized by the activation of fibroblasts in the renal interstitial and deposition of extracellular matrix (ECM) components, known as renal fibrosis, which leads to the loss of normal renal structure as well as renal function, and ultimately leading to renal failure requiring renal replacement therapy (Zhou et al., 2016). Our research group has conducted a number of studies on the role and mechanism of EZH2 in AKI and CKD, and published a number of papers and relevant reviews.

**FIGURE 1:**
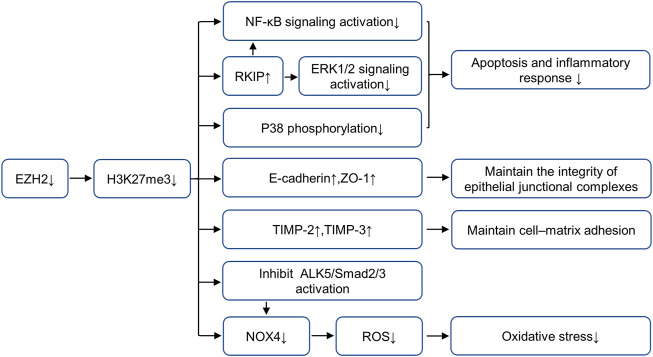
Role and mechanism of EZH2 inhibition in AKI. EZH2 inhibition plays a protective role in acute kidney injury by preserving the expression of RKIP, E-cadherin, TIMP-2 and TIMP-3, and repressing the activation of multiple fibrosis and inflammatory signaling pathways, including NF-κB, ERK1/2, p38 and ALK5/Smad2/3. AbbreviationsAKI, acute kidney injury; EZH2, enhancer of zeste homolog 2; TIMP-2, metalloproteinase-2; TIMP-3, metalloproteinase-3; RKIP, raf kinase inhibitor protein; NF-κB, nuclear factor-κB; ERK, extracellular signal-regulated kinase.

Previous studies of our research group (Zhou et al., 2018a; Ni et al., 2019) have proved that in mice models of AKI (including folic acid, IR and cisplatin-induced AKI models), EZH2 and its specific substrate H3K27me3 expressions are increased in the damaged kidney, blockade of EZH2 with 3-DZNeP (an inhibitor of S-adenosylhomocysteine hydrolase that inhibits the activity of EZH2) can reduce kidney injury and protect renal function. To further explore the mechanism of EZH2 regulating kidney injury in AKI, we find that EZH2 may mediate the transcription of E-cadherin, tissue inhibitor of metalloproteinase (TIMP)-2, tissue inhibitor of metalloproteinase (TIMP)-3 and Raf kinase inhibitor protein (RKIP) to regulate their expressions, thus destroying the adhesion and connection between cells and cells or cells and matrix, increasing apoptosis and inflammatory response (Zhou et al., 2018b; Ni et al., 2019). Several latest research results support this argument. ZLD1039 (a selective EZH2 inhibitor) effectively inhibits the expression of EZH2 and H3K27me3, and blocked the upregulation of RKIP and activation of nuclear factor-κB (NF-κB) p65 signaling pathway, thus alleviating inflammation in cisplatin-induced AKI (Wen et al., 2021). This research further demonstrates that H3K27me3 binds to p65 (Rela) and RKIP (PEBP1) promoter regions by CHIP assay, but EZH2 does not directly recruit to these two promoter regions, suggesting that the inhibition of EZH2 on p65 and RKIP expression mainly depends on its methyltransferase activity (Wen et al., 2021). Moreover, EZH2-mediated H3K27 methylation attenuates the expression of TIMP-2 and TIMP-3 by inducing promoter DNA methylation in prostate cancer (Shin and Kim, 2012) while the silencing of E-cadherin occurs through H3K27 methylation without DNA methylation in non-small-cell lung carcinoma (Jia et al., 2021). However, the regulatory mechanism of EZH2 on E-cadherin and TIMPs is still unclear in AKI and requires further study. In addition, EZH2 can reduce kidney injury via regulating oxidative stress and pyroptosis in IR-induced AKI (Liu et al., 2020). On one hand, EZH2 can regulate Nox4 activity through the ALK5/SMad2/3 pathway, and on the other hand, it acts on the Nox4 promoter region to regulate its transcription (Liu et al., 2020). EZH2 can also increase apoptosis and inflammation through P38 signaling pathway in IR-induced AKI. The role and mechanisms of EZH2 in AKI are summarized in Figure 1.

About 1/4 of AKI patients will evolve into CKD, and eventually enter end-stage renal disease, requiring kidney replacement therapy (Noble et al., 2020). In unilateral ureteral obstruction (UUO) mice model (Zhou et al., 2016), EZH2 mediates myofibroblast activation through the TGF-β/Smad3 signaling pathway, but EZH2-mediated H3K27me3 does not bind to the TGFβ-RI promoter region, suggesting that EZH2 may regulate gene expression through histone methyltransferase independent mechanisms. In addition, EZH2 further mediates the phosphorylation of epidermal growth factor receptor (EGFR) and platelet-derived growth factor receptor (PDGFR) by regulating the expression of tensin homolog deleted on chromosome 10 (PTEN), and the activation of its downstream signaling pathways signal transducer and activator of transcription 3 (STAT3) and ERK1/2 to regulate kidney fibrosis (Zhou et al., 2016). EZH2 can also inhibit E-cadherin expression and promotes epithelial-mesenchymal transition (EMT) by increasing snail-1 level through activation of Akt/β-catenin signaling pathway (Zhou et al., 2018a), and the regulation of EZH2 on Akt pathway is also mediated by PTEN. In non-small cell lung cancer, EZH2-mediated H3K27me3 has been demonstrated to bind directly to the promoter region of PTEN (Zang et al., 2020), but how EZH2 regulates PTEN in CKD requiring further research.

Hyperuricemia and lupus nephritis (LN) are two important causes of CKD, and overexpression of EZH2 and H3K27me3 is detected in those injury kidneys (Rohraff et al., 2019; Shi et al., 2019). In mouse model of hyperuricemia, 3-DZNeP treatment reduces serum uric acid level and attenuates renal injury and fibrosis through multiple profibrotic signaling pathways including TGF-β/Smad3 and EGFR/ERK1/2, and reduces the release of proinflammation chemokines/cytokines via NF-κB signaling pathways (Shi et al., 2019). Lupus nephritis (LN) is the complications of Systemic lupus erythematosus, estimating to affect up to 60% of patients with lupus (Koutsokeras and Healy, 2014; Zhu et al., 2016). In CD4^+^ T cells of patients with lupus, upregulation of EZH2 and H3K27me3 alters the methylation status, and overexpression of EZH2 promotes CD4^+^ T cells adhesion to endothelial cells through demethylation and upregulation of JAM-A, thus increasing disease activity in lupus (Tsou et al., 2018). Blockade of EZH2 can also inhibit the production of cytokine and chemokine in LN (Rohraff et al., 2019). Collectively, in obstructive nephropathy, hyperuricemic nephropathy and lupus nephritis, EZH2 inhibition has a protective effect on the kidney and can delay the progression of the disease. However, the role of EZH2 in diabetic nephropathy is still controversial.

Diabetic Nephropathy (DN) is one of the most common chronic complications of diabetes, and its pathological changes are mainly manifested as mesangial hyperplasia, thickening of basement membrane, capillary cavity occlusion, structural disorder of podocytes, and reduced number of podocytes (Tung et al., 2018). In recent researches, the expression of EZH2 is closely related to podocyte injury. In diabetic rats, H3K27me3 is enriched in the promoter region of Pax6 gene, and inhibition of EZH2 can further down-regulate H3K27me3 at Pax6 promoter and increase the expression of Pax6, thus upregulated Pax6 binds to the thioredoxin interacting protein (TxnIP) promoter and increases the expression of TxnIP in podocytes, causing podocyte damage and renal dysfunction (Siddiqi et al., 2016). The protective role of H3K27me3 in glomerular podocyte is also supported by other findings that inhibition of H3K27me3 demethylases Jmjd3 and UTX increasing podocyte H3K27me3 level and alleviating glomerular disease (Majumder et al., 2018). In addition to podocytes, EZH2 also has protective effect on mesangial cells in DN (Zeng et al., 2018; Jia et al., 2019). Inhibition or deletion of EZH2 leads to up-regulation of fibrotic and inflammatory genes in mesangial cells and increases glomerular dysfunction (Jia et al., 2019). However, the role of EZH2 in diabetic nephropathy is controversial. Blocking EZH2 can attenuate podocyte injury in diabetic nephropathy via mediating β-catenin inactivation (Wan et al., 2017). EZH2 silencing or inhibition can also rescue SIRT1 expression and block ROS accumulation, thus suppressing EMT and fibrogenesis (Zeng et al., 2018).

In conclusion, EZH2 mainly through two action modes to regulate the development of AKI and CKD: 1) By catalyzing H3K27 trimethylation in the nucleus to silence numerous target genes, thereby affecting the expression or phosphorylation of target proteins and the activation of downstream signaling pathways (Wen et al., 2021; Liu et al., 2020); 2) EZH2 can also be localized to the cytoplasm and methylate non-histone proteins such as STAT3 (Zhou et al., 2016) and β-catenin (Wan et al., 2017). The role and mechanism of EZH2 in different chronic kidney diseases is summarized in Table 1. Blockade of EZH2 plays a protective role in obstructive nephropathy, hyperuricemic nephropathy and lupus nephritis, attenuating renal fibrosis and alleviating renal dysfunction. In contrast, EZH2 may play a protective role in DN and reduces renal dysfunction caused by continuous high glucose stimulation. On one hand, the pathogenesis of DN involves a variety of cell types, and various intracellular environment affects the gene regulation of EZH2 (Brasacchio et al., 2009); Secondly, in response to sustained injury, the role of EZH2-regulated signaling pathways changes, such as the EGFR signaling pathway, which is essential for the initial renal tubular cell repair response, but its continued activation will eventually lead to renal fibrosis (Tang et al., 2013); finally, the balance of antagonism between proteins also affects the expression of EZH2 (Wan et al., 2017).

**TABLE 1:** EZH2 inhibition on various chronic kidney diseases.

Kidney Disease	Mechanisms	Effects	References
UUO	Inhibition of several profibrotic signaling pathways (TGF-β/Smad3, EGFR&PDGFR, STAT3, ERK1/2, Akt/β-catenin)	Attenuate fibrosis	Zhou et al. (2018a) Zhou et al. (2016)
Hyperuricemia	Inhibition of profibrotic signaling pathway and proinflammation chemokines/cytokines	Reduce the level of serum uric acid and attenuate fibrosis	Shi et al. (2019)
Lupus nephritis	Reducing T cell adhesion via inhibiting the demethylation and upregulation of JAM-A; Inhibition cytokine and chemokine production	Inhibit lupus activity	Tsou et al. (2018) Rohraff et al. (2019)
Diabetic Nephropathy	Increases Pax6 and TxnIP expression in podocyte	podocyte injury and renal dysfunction	Siddiqi et al. (2016)
	up-regulation of fibrotic and inflammatory genes in mesangial cells	glomerular dysfunction	Jia et al. (2019)
	reduce ROS accumulation in HK2 cells	Reduce renal tubular injury	Zeng et al. (2018)
	repression of β-catenin pathway	Ameliorate’ podocyte injury	Wan et al. (2017)

Abbreviations: UUO, unilateral ureteral obstruction; EGFR, epidermal growth factor receptor; PDGFR, platelet-derived growth factor receptor; NF-κB, nuclear factor-κB; PTEN, tensin homolog deleted on chromosome 10; STAT3, signal transducer and activator of transcription 3; TxnIP, thioredoxin interacting protein; ERK, extracellular signal-regulated kinase.

### Disruptor Of Telomeric Silencing 1-Like Protein

**FIGURE 2:**
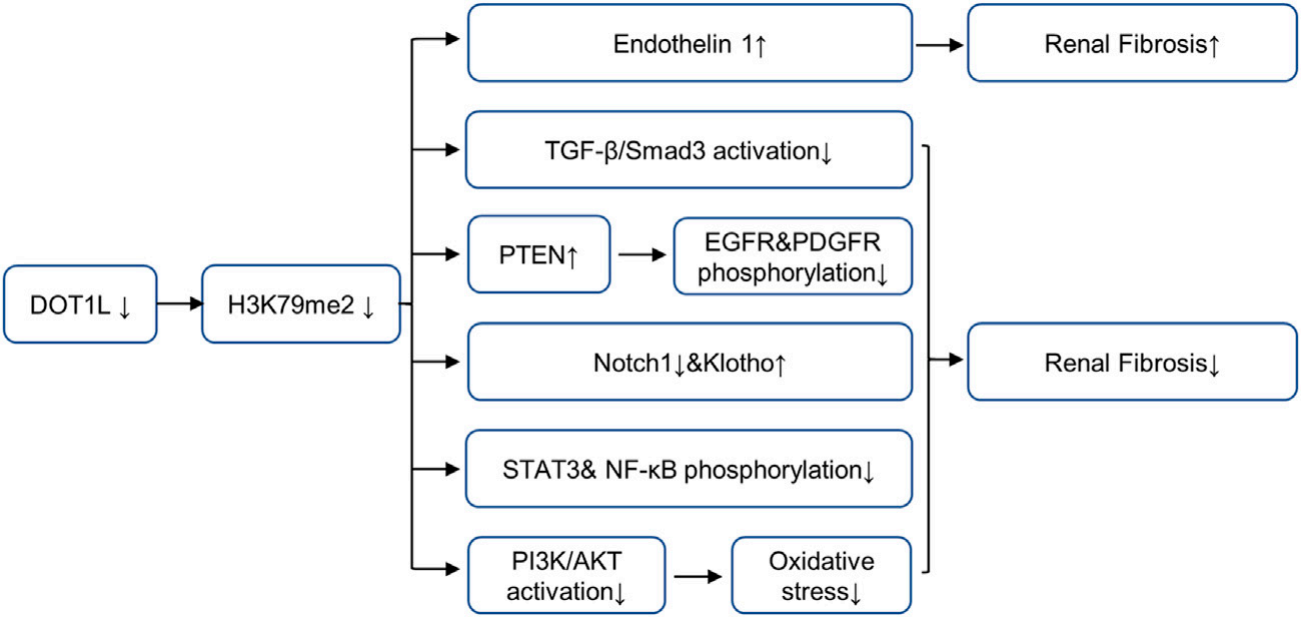
Role and mechanism of DOT1L inhibition in renal fibrosis. Inhibition of DOT1L promotes renal fibrosis by up-regulating ET1. In contrast, Inhibition of DOT1L alleviates renal fibrosis by inhibiting multiple fibrosis pathways (including Smad3, EGFR, PDGFR and NF-κB pathway), up-regulating the expressions of renal protective factors such as PTEN, Klotho and Smad7,2, and preventing the generation of ROS via the PI3K/Akt pathway. AbbreviationsDOT1L, disruptor of telomeric silencing 1-like protein; EGFR, epidermal growth factor receptor; PDGFR, platelet-derived growth factor receptor; NF-κB, nuclear factor-κB; PTEN, tensin homolog deleted on chromosome 10; STAT3, signal transducer and activator of transcription 3; ET1, endothelin 1.

The epigenetic regulator Dot1L is the only known histone H3K79 methyltransferase, which contains four conserved sequence motifs of class I SAM-dependent methyltransferases instead of the classical SET domain (Wang et al., 2021). Dot1L is widely expressed in rats and humans and can specifically catalyze mono-methylation, di-methylation, and tri-methylation of H3K79 (Yang et al., 2019). H3K79 methylation is widely believed to be a biomarker of active gene transcription (Anglin and Song, 2013). Studies have shown that Dot1L plays a conserved role in organism development, conditional inactivation of Dot1L causes congenital renal dysplasia (Wang et al., 2021). Dot1L conditional knockout mice showed endothelin 1 (ET1) upregulation 2 months after delivery (Wu et al., 2013). Further studies in UUO and DN mice models show that Dot1L and HDAC2 counterbalance to regulate the gene transcription encoding endothelin 1 (Edn1) by modulating H3K79 di-methylation and H3 acetylation associated with the Edn1 promoter, and the loss of DOT1L promotes renal fibrosis by up-regulating ET1 (Zhang et al., 2020). Moreover, both αENaC and Edn1 are aldosterone target genes, Dot1L has been confirmed to inhibit αENaC by recombining with Af9 at the αENaC promoter and promoting methylation of H3K79me2; aldosterone can relieve the repression via inhibiting the formation of this complex (Zhang et al., 2006a; Zhang et al., 2006b). This mechanism may also applicable to Edn1. In contrast, in UUO models, blocking DOT1L with EPZ5676 alleviates renal fibrosis by inhibiting multiple fibrosis pathways (including Smad3, EGFR, PDGFR and NF-κB pathway), up-regulating the expressions of renal protective factors such as PTEN, Klotho and Smad7,2 (Liu H. et al., 2019). In IR-induced AKI models, blocking DOT1L with EPZ004777 could alleviate renal fibrosis by preventing the generation of ROS via the PI3K/AKT pathway (Yang et al., 2019). The controversy over the role of DOT1L in different renal disease models are shown in Figure 2, suggesting that DOT1L affects kidney through multiple pathways, which requires further study. Due to the complexity of the occurrence and development mechanism of CKD, the balance of the regulation of DOT1L on different target proteins and profibrotic signaling pathway will affect the progression of CKD, and the regulation of DOT1L may be affected by the action of other methyltransferases or acetylases.

### G9a

Histone lysine methyltransferase G9a consists of a SET catalytic domain, an ankyrin repeat, and a glutamate- and cysteine-rich region at the N-terminus (Zhang et al., 2016), and is a key enzyme for H3K9 mono- and di-methylation (me1 and me2) (Tachibana et al., 2002). G9a has recently become the focus of attention due to its important role in promoting tumorigenesis and metastasis (Yuan et al., 2013; Chang et al., 2015). Elevated G9a expression is detected in tumor tissues and adjacent tissues from 80 patients with renal cell carcinoma (RCC) (Li et al., 2021). UNC0638, a specific G9a inhibitor, significantly inhibits the proliferation, migration and invasion of renal carcinoma *in vitro* and *in vivo* (Li et al., 2021). In addition, SPINK5 is demonstrated to be one of the downstream target genes of G9a, and G9a promotes the development of renal cancer through epigenetic silencing of tumor suppressor gene SPINK5 (Li et al., 2021). BIX01294 is a potent and selective G9a inhibitor that modulates the methylation status of H3K9 (Vedadi et al., 2011). *In vitro*, BIX01294 has been shown to facilitates TRAIL-mediated apoptosis of human renal carcinoma Caki cells by downregulating survivin and upregulating DR5 expression, suggesting that BIX01294 could be a therapeutic strategy to overcome TRAIL resistance in cancer cells (Woo et al., 2018). G9a also plays an important role in AKI and renal fibrosis. In IR-induced AKI model, IR injury resulted in a rapid increase in G9a accompanied by down-regulation of Sirt1 (Liu B. et al., 2021). Further investigation showed that G9a interacts with chromobox homolog 1 (CBX1) to catalyze H3K9 demethylation and ultimately inhibit Sirt1 transcription (Liu H. et al., 2021), suggesting that the G9A-SIRT1 axis may be a promising therapeutic target in an epigenetic manner. In UUO-induced renal fibrosis model, TGF-β1 induces G9a expression via Smad3 activation, and BIX01294 inhibits G9a-mediated H3K9me1 and attenuates renal fibrosis and retains klotho expression (Irifuku et al., 2016). In addition, the expression of G9a was correlated with renal tubulointerstitial fibrosis and renal Klotho expression in human renal biopsy specimens (Irifuku et al., 2016). These results suggest that G9a plays an important role in the progression of renal fibrosis.

### Emerging Targets

The SMYD family of lysine methyltransferases consists of five members (SMYD1-5), and these enzymes have various functions in cancer (Tracy et al., 2018). SMYD2 mediates histone H3 lysine 36 trimethylation (H3K36me3) and acts as a regulator of tumorigenesis (Liu H. et al., 2021). SMYD2 has been reported to be highly expressed in renal epithelial cells and tissues from Pkd1-knockout mice and human autosomal dominant polycystic kidney disease patients (Li et al., 2017). In addition, SMYD2 is highly expressed in the kidney of UUO mice models, and inhibition of SMYD2 with AZ505 reduces the phosphorylation of several fibrosis signaling pathways including Smad3, ERK1/2, Akt, STAT3 and NF-κB, and up-regulates Smad7 *in vivo* and *in vitro* (Liu L. et al., 2021).

SET domain containing lysine methyltransferase 7 (SETD7) belongs to the evolutionarily conserved Su (var) enhancer of zeste and trithorax domain family, which is originally characterized as a mono-methyltransferase of lysine 4 on histone H3 (Liu L. et al., 2019). SETD7 expression is positively correlated with the degree of interstitial fibrosis in human renal biopsy specimens from patients with IgA and membranous nephropathy. This conclusion has been verified in animal models. In UUO mice, inhibition of SETD7 expression can reduce renal fibrosis (Sasaki et al., 2016). In addition, previous studies have shown that SETD7 contributes to AKI (Sasaki et al., 2016) and renal fibrosis in diabetic mice (Goru et al., 2016; Sasaki et al., 2016). SETD7 mediates M2 macrophages-myofibroblasts transition, bone marrow-derived myofibroblasts activation, and inflammation response in the development of renal fibrosis (Liu B. et al., 2021).

### Arginine Methyltransferases

**FIGURE 3:**
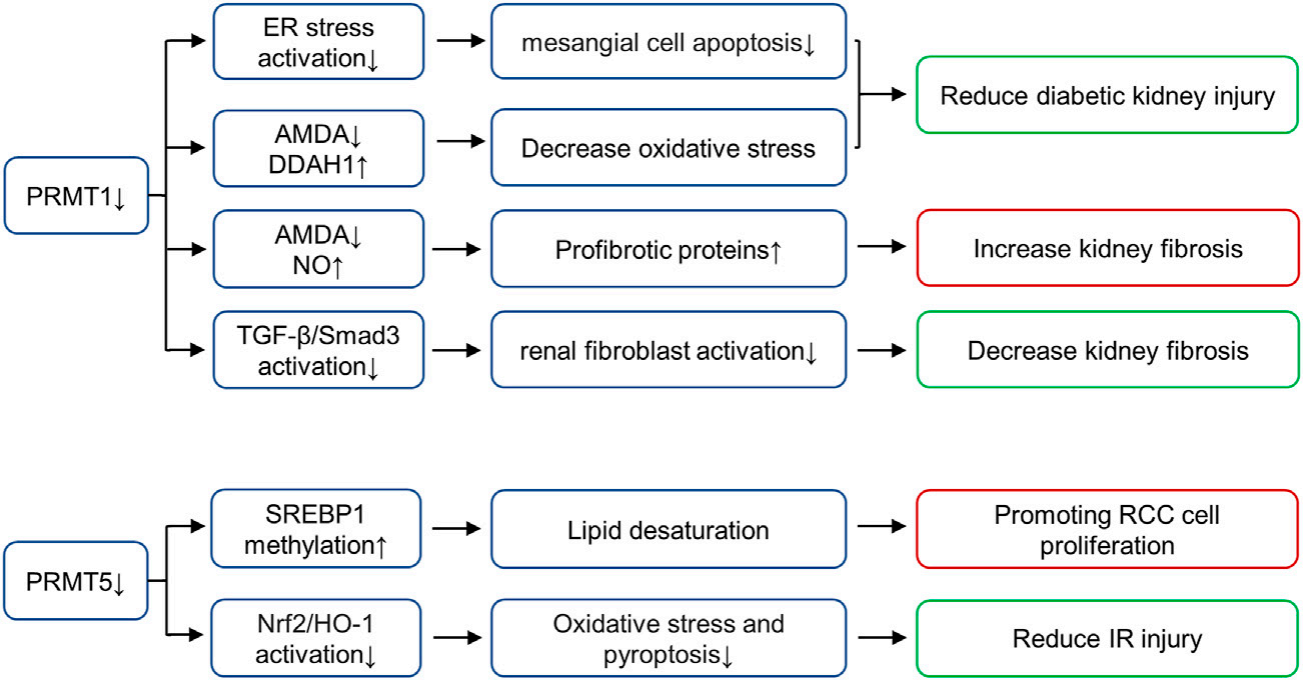
Role of histone arginine methyltransferases in various renal diseases. PRMT1 increases oxidative stress and promotes diabetic kidney injury by regulating the metabolic axis of PRMT1-AMDA-DDAH1. PRMT1 overexpression promotes endoplasmic reticulum (ER), thus inducing mesangial cell apoptosis in DN, PRMT1 knockdown reduces the injury. Inhibition of PRMT1 can inhibit the activation of TGF-β/Smad3 signaling pathway and alleviate renal fibrosis. PRMT1 has also been shown to affect NO production and reduce renal fibrosis through AMDA, and inhibition of PRMT1 can increase this pathological progression. PRMT5 interacts with LINC01138 to enhance protein stability and methylation of SREBP1, further mediating lipid desaturation and promoting RCC cell proliferation. PRMT5 is also involved in ischemia- and hypoxia-induced oxidative stress and pyroptosis via activation of the Nrf2/HO-1 signal pathway. AbbreviationsPRMT, protein arginine methyltransferases; DDAH, dimethylarginine dimethylaminohydrolase; SREBP1, sterol regulatory element-binding protein 1; RCC, renal cell carcinoma; Nrf2, NF-E2-related factor; HO-1, heme oxygenase-1; ADMA, asymmetrical di-methylarginine.

Arginine methylation mediated by nine members of the PRMT family is an important epigenetic mechanism. The PRMTs can be classified as type I (PRMT1-4, PRMT6, PRMT8), II (PRMT5 and PRMT9), III (PRMT7) or IV (enzymes) depending on the specific catalytic activities (Zhang and Zhuang, 2020). All type PRMT enzymes can catalyze arginine mono-methylation, type I PRMT catalyzes asymmetrical di-methylarginine (ADMA), while type II catalyzes symmetric di-methylarginine (SDMA) (Bedford and Clarke, 2009; Blanc and Richard, 2017). Among all PRMT members, PRMT1 and PRMT5 mainly affect arginine methylation levels in mammalian cells (Zhang and Zhuang, 2020). PRMT1 is responsible for up to 85% generation of AMDA and PRMT5 is the major type II enzyme catalyzing SDMA (Stopa et al., 2015; Li et al., 2019). In the following paragraphs, we will summarize the role of these two enzymes in kidney disease (Figure 3).

### PRMT1

PRMT1 known substrates including histone substrates (H4R3me2a and H2AR3me2a) and non-histone substrates (RBM15, Heat-shock protein of 70 kDa, FAM98A, Class II transactivator, SMAD7, c-FLIPL, FMS-like receptor tyrosine kinase-3, C/EBPα and MyoD) (Zhang and Zhuang, 2020). As the main subtype of type I PRMT, PRMT1 performs more than 80% of PRMT activity in mammalian cells and is distributed in multiple organs, including heart, kidney, liver and lung (Yu et al., 2009). In animal models and humans, elevated ADMA concentrations have been observed in diabetes, and ADMA is considered a risk factor for diabetic nephropathy (Lin et al., 2002). In diabetic rat models, the expression of PRMT1 and AMDA increases, while the expression of type 1 dimethylarginine dimethylaminohydrolase (DDAH1) decreases. PRMT1 increases oxidative stress and promotes diabetic kidney injury by regulating the metabolic axis of PRMT1-AMDA-DDAH1 (Mei et al., 2021). In addition, lipotoxicity induced PRMT1 overexpression and promoted endoplasmic reticulum (ER), thus inducing mesangial cell apoptosis in DN, PRMT1 knockdown reduces the injury (Park et al., 2017). In UUO mice models, Previous studies in our group find that PRMT1 and H4R3me2a are up-regulated in the UUO mice models, and inhibition of PRMT1 via AMI-1 can inhibit the activation of TGF-β/Smad3 signaling pathway and alleviate renal fibrosis (Zhu et al., 2020). However, the role of PRMT1 in UUO model is controversial. PRMT1 has also been shown to affect NO production and reduce renal fibrosis through AMDA, and inhibition of PRMT1 with PT1001B can increase this pathological progression (Wu et al., 2019). The difference in the results of these two studies in UUO models may due to the non-specificity of the inhibitor (which acts on other biological targets except PRMT1), the use of PRMT1 knockout mice may be helped in further research. In general, we find that PRMT1 plays a role in kidney through ADMA synthesis or epigenetic gene regulation mediated by histone modification or even non-histone modification mediated transcription factors, and its role in renal diseases deserves further study.

### PRMT5

PRMT5 is the major type II enzyme catalyzing SDMA involved in pathways known to be dysregulated in cancer, including transcription, cell signaling, mRNA translation and DNA damage (Stopa et al., 2015). PRMT5 known substrates including histone substrates (H2AR3me2s, H3R2me2s, H3R8me2s and H4R3me2s) and non-histone substrates (PTEN, Zinc finger protein 326, Tyrosyl-DNA phosphodiesterases, Tripartite motif-containing protein 21, Sterol regulatory element-binding protein 1, F-box/WD repeat-containing protein 7, Forkhead box P3, Protein kinase B and SIRT7) (Zhang and Zhuang, 2020). *In vitro*, PRMT5 interacts with LINC01138 to enhance protein stability and methylation of SREBP1, further mediating lipid desaturation and promoting RCC cell proliferation (Zhang et al., 2018). In addition, the expression of PRMT5 is enhanced in the renal tubular epithelium of animals subjected to IR injury (Braun et al., 2004). Inhibition of PRMT5 by EPZ represses the activation of NF-E2-related factor/heme oxygenase-1 signaling pathway, reducing ROS production, thus alleviating oxidative stress and pyroptosis (Diao et al., 2019). At present, a variety of PRMT5 inhibitors are in the stage of clinical trials to be used for the treatment of solid tumors, including GSK3326595, JNJ-64619178 and PF-06939999 (Rugo et al., 2020). However, PRMT5 has not been put into clinical trials for the treatment of kidney diseases, which requires exploration and support of various experimental theories.

## Conclusion and Perspectives

At present, many histone Lysine methyltransferases or histone arginine methyltransferases inhibitors have made gratifying progress in animal models of different kidney diseases. It turns out that modulating epigenetic targets in the treatment of kidney disease is a rich research area, and a large number of new epigenetic modifiers are being developed. Some of these have shown early signs of activity in clinical trials (Table 2). Histone methyltransferase inhibitors are a promising new approach. However, there are still many unanswered questions about the best way to use these drugs, including patient selection, identification of biomarkers, correct drug combinations and improved drug selectivity. The combination of epigenetic modifiers with immunotherapy, standard chemotherapy, radiotherapy, angiogenesis inhibition or hormone therapy are also an active area of research. The results of various ongoing basic and clinical trials will no doubt provide answers to some of these questions and pave the way for new treatments for patients with unmet needs.

**TABLE 2:** Overview of methyltransferase inhibitors under clinical investigation.

Target Class	Clinical Compounds	Indications in Clinical Studies	Ref
EZH2	Tazemetostat	DLBCL, FL, and SNF5/INI-1/SMARCB1 genetically defined solid tumors	Kung et al. (2018)
	CPI-0209	advanced tumors	Lakhani et al. (2021)
	CPI-1205	DLBCL, FL, and SNF5/INI-1/SMARCB1 genetically defined solid tumors	Kung et al. (2018)
DOT1L	Pinometostat	MLL-rearranged leukemias	Bon et al. (2021)
PRMT1	GSK3368715	DLBCL, solid tumors	Bon et al. (2021)
PRMT5	GSK3326595	MDS, AML, olid tumors, NHL	Lin and Luengo, (2019)
	JNJ-64619178	B cell NHL, solid tumors	Rugo et al. (2020)
	PF-06939999	Advanced or metastatic solid tumors	Rugo et al. (2020)

Abbreviations: EZH2, enhancer of zeste homolog 2; DOT1L, disruptor of telomeric silencing 1-like protein; PRMT, protein arginine methyltransferases; MLL, mixed-lineage leukemia; DLBCL, large B-cell lymphoma; FL, follicular lymphoma; AML, acute myeloid lymphoma; MDS, myelodysplastic syndrome; NHL, non-Hodgkin’s lymphoma.
